# Perfectionism and fear of failure, according to sporting experience. A referee handball study

**DOI:** 10.3389/fpsyg.2025.1542416

**Published:** 2025-05-16

**Authors:** Manuel Gómez-López, David Manzano-Sánchez, Carla Chicau Borrego, Juan González-Hernández

**Affiliations:** ^1^Faculty of Sport Sciences, University of Murcia, San Javier, Murcia, Spain; ^2^Faculty of Education Sciences, University of Almería, Almería, Spain; ^3^Sport Sciences School of Rio Maior, Polytechnic Institute of Santarém, SPRINT - Sport Physical activity and health Research & Innovation Center, Rio Maior, Portugal; ^4^Faculty of Psychology, University of Granada, Granada, Spain

**Keywords:** experience, perfectionism, concern over mistakes, referee, fear

## Abstract

**Introduction:**

Fear of failure is a motive to avoid failure in evaluative situations based on anticipatory shame upon failure. Perfectionism is a personality disposition characterized by striving for flawlessness and setting exceedingly high standards of performance accompanied by overly critical evaluations of one’s behavior. The goal of the study was to describe the effects of previous sports experiences that handball referees had, related to perfectionism and fear of making mistakes.

**Methods:**

Data were collected from 120 referees (93 males, 77.5%; 27 females, 22.5%), ranging in age from 14 to 62 years (*M* = 28.12; SD = 11.99), using purposive sampling methods. The Performance Failure Assessment Inventory and the Multidimensional Inventory of Perfectionism in Sport were used. Data were analyzed using differential, correlational and regression analysis.

**Results:**

Results showed that both perfectionistic tendencies and fear of failure provide interaction effects on fear of failure. Differences in perfectionist tendencies according to gender (women) and in favor of referees who were coaches or handball players before becoming referees.

**Discussion:**

Interventions can be developed to help reduce Fear of Failure and Perfectionism.

## 1 Introduction

Although traditionally in the field of sport the figure of the referee, as an object of study, has been in the background behind athletes (González-Oya and Dosil, 2004), it is impossible not to consider their importance for the proper development of sports activity. Proof of this is that it is currently an emerging and relevant field of research (Philippe et al., 2009; Raab et al., 2019; Slack et al., 2012). Refereeing is a sporting and, sometimes, professional activity where psychological variables play a fundamental role (Guillén, 2003; Weinberg and Richardson, 1990).

In highly competitive sports contexts, sport becomes a means in which the referees often feel incompetent because of their public exposure limits the control of decisions and situations. Referees must take important and influential decisions during the match, experiencing a psychological reactivity (e.g., fear of failure) under external evaluation of their performance and success by different external agents (e.g., public, athletes, media) (Sagar et al., 2007).

Fear of failure could be described as a stable tendency to anticipate embarrassment and humiliation after failure (Atkinson, 1957; Conroy et al., 2001, 2002). Feelings that provoke insecurity, anxiety and stress, leading to avoidance behaviors (e.g., excessive exercise) and impacting on their sport performance (Correia and Rosado, 2018; Gómez-López et al., 2020). Ogunsanya and Rasheed (2019) demonstrated that fear of failure, stress alter the physical condition of referees, contributing significantly negatively to decision making in competition in a sample of 101 soccer referees.

Experience is a factor that could modulate the emotional states of athletes, because it seems to be positively related to the mastery of diverse psychological responses (Muñoz-Arjona and Castillo-Rodríguez, 2020), with the most experienced athletes having lower scores in negative psychological responses (e.g., melancholy, anxiety, stress) and higher in positive ones (e.g., motivation, confidence, concentration; Castillo-Rodríguez et al., 2023; Hanton et al., 2008).

Studies have shown that experienced referees show better psychological characteristics (e.g., emotions under pressure) and can concentrate with greater intensity (Muñoz-Arjona et al., 2022; Samulski and Noce, 2003). Furthermore, studies showed that there is a negative relationship between the degree of experience and anxiety and a positive relationship with self-confidence and self-esteem (Castillo-Rodríguez et al., 2023; Hanton et al., 2008; Muñoz-Arjona and Castillo-Rodríguez, 2020; Muñoz-Arjona et al., 2022) and self-efficacy (Aguilar et al., 2021; Alsharji et al., 2019). Alsharji et al. (2019) how self-efficacy can be used to differentiate expert referees from less experienced ones in a sample of handball referees.

Aguilar et al. (2021), Soccer referees at a national level with an experience greater to 8 years, had higher levels of self-efficacy than those with less experience. On the other hand, Pedrosa and García-Cueto (2016) found that more experienced referees presented a higher risk of suffering from burnout syndrome. Aguirre-Loaiza et al. (2020), did not find psychological characteristics related to sports performance between head referees and assistant referees, depending on refereeing procedure or work experience.

The literature has shown that competitive sport is considered an unbeatable field for the study of perfectionism, both because of its nature for the achievement of personal objectives or standards (Dunn et al., 2002) and because of the values of excellence that are pursued in it to obtain optimal performance. Perfectionism is understood as a disposition of the personality characterized by an excessive level of demand in performance and accompanied by a tendency to excessively critical self-evaluation of one’s behavior (Egan et al., 2011; Flett and Hewitt, 2002; Frost et al., 1990).

Traditionally, from clinical contexts of psychology and psychiatry, perfectionism has been considered an essentially negative or maladaptive and dysfunctional aspect of personality, understood as a risk factor or process in the etiology and maintenance of other psychological disorders (Egan et al., 2011; Flett and Hewitt, 2002).

A perfectionist person tends to set goals that are too high and unrealistic, adhere rigidly to them and value themself in terms of achieving them (Hewitt and Flett, 1991; Frost et al., 1990; Gustafsson et al., 2023), and agonisingly experiences this process by responding psychologically.

According to some authors, perfectionist athletes fear failure and mistakes to such an extent that their sports enjoyment and performance are reduced and hindered (Correia, 2018; Frost and Henderson, 1991; Gustafsson et al., 2017). Fear of failure is a subjective emotion, which has environmental antecedents -interpersonal perfectionism-, and affective consequences (Pineda-Espejel et al., 2019). Moreover, fear of failure has been associated with maladaptive perfectionist behavior (e.g., self-discriminations, unrealistic thoughts) and a primary motivation underlying perfectionism (e.g., commitment) (Correia et al., 2018; Sagar and Stoeber, 2009).

### 1.1 Perfectionism in sports referees. More pressure to live with mistakes

The literature that has delved into the relationships between perfectionist tendencies in sport contexts has not considered samples of referees and judges. Being under pressure, seeking the best possible action at any given moment, reducing the occurrence of failure and exposure to criticism are processes quite common to those of any other sport figure or performance context.

Under this premises, self-oriented perfectionism, which involves the tendency to set unrealistic standards for oneself and to harshly evaluate and criticize one’s behavior as a result of an impulse to achieve perfection and avoid failure (Hewitt and Flett, 1991), showed positive correlations with four of the five aversive causes of fear of failure (in this case the correlation with fear of having an uncertain future was not significant). However, in regression analyses, only socially prescribed perfectionism was associated with fear of important others losing interest and with fear of upsetting important others (these were classified as aversive interpersonal consequences of failure), while self-oriented perfectionism showed no association. According to these authors, these results suggest that the perfectionist concerns dimension of perfectionism shows close links to the fear of failure, but not to the perfectionist efforts dimension, as the study indicated that the fear of failure was more likely to motivate socially prescribed perfectionism than any of the other forms of perfectionism.

Similarly, socially prescribed perfectionism was especially linked to beliefs about the aversive interpersonal consequences of failure. Another study, conducted by Kaye et al. (2008), confirmed these relationships, examining how perfectionist personal standards (the perfectionism marker variable of individual expectations) and perfectionist preoccupation with mistakes (perfectionism’s marker variable of evaluative concerns) were related to the five aversive causes of fear of failure. Stoeber and Becker (2008) investigated the relationships between perfectionism, hope of success, and fear of failure in female soccer players. The results of the research showed a positive correlation between perfectionist concerns and fear of failure.

On the other hand, Sagar and Stoeber (2009) demonstrated with a sample of athletes that the fear of experiencing shame is fundamental in the relationship between fear of failure and perfectionism and that perfectionist concern about mistakes and coach pressure are aspects of perfectionism that predict the fear of experiencing shame and negative affect after failure. While perfectionist efforts predicted a lower fear of experiencing shame, perfectionist concerns predicted greater fears of failure in the five aversive causes of fear of failure.

Later, the results of the study carried out by Correia et al. (2018) also with athletes, reflected strong correlations between perfectionism and fear of failure, specifically in the dimension of concern for mistakes, reflecting the most maladaptive aspects of the perfectionist dimension. In particular, the dimension of concern for errors has a significant influence on the dimension of fear of failure, followed by doubts about actions. Therefore, both perfectionist dimensions are recognized as the central aspects of perfectionism and predictors of all dimensions of fear of failure in athletes.

As has been shown, the relationship between perfectionism and fear of failure in sports is evident and evidenced by results indicating that aspects of both dimensions of perfectionism (i.e., perfectionist efforts and perfectionist concerns) show positive correlations with fear of failure, suggesting that fear of failure is associated with all aspects of perfectionism (Frost and Henderson, 1991; Kaye et al., 2008; Sunkarapalli and Agarwal, 2017; Stoeber and Becker, 2008). However, a more recent and detailed examination reveals a predominantly positive correlation between aspects of the evaluative concerns dimension of perfectionism and fear of failure (Correia et al., 2018).

Based on all that has been described so far and the present work, the study aims to describe the effects of previous sports experiences on the two facets of individual perfectionism (striving for perfection, and negative reactions to imperfection) and the fear of making mistakes in handball referees.

## 2 Method

### 2.1 Design and participants

Given the characteristics of the study and the sample, a descriptive, non-randomized, exploratory cross-sectional design was used. A total of 120 handball referees of different categories participated (93 men, 77.5%; 27 women, 22.5%). The age range was between 14 and 62 years (*M* = 28.12; *SD* = 11.99). Table 1 shows the percentages of the different sociodemographic and refereeing-related categorical variables considered in this study.

**Table 1:** Sociodemographic data of handball referees.

		*N*	%	Dispersion
Gender	Male	93	77.5%	37.90 (<0.32)*
	Female	27	22.5%	
Category where you referee	Grassroot sport	33	27.5%	71.64 (0.02)
	National	66	55.0%	
	Professional	21	17.5%	
Experience as a referee	<10 years	95	79.2%	21.61 (0.01)
	>10 years	25	20.8%	
Previous handball experience	Only player	70	58.3%	102.33 (0.41)*
	Player and coach	47	39.2%	
	Never in other sporting roles	3	2.5%	
Referee of another sport	No	109	90.8%	81.98 (0.03)
	Yes	11	9.2%	
Category promotion	No	63	52.5%	0.53 (0.02)
	Yes	57	47.5%	

*If *p* > 0.05.

### 2.2 Measures

Fear of failure. To measure fear of failure, the long version of the Performance Failure Appraisal Inventory (PFAI; Conroy et al., 2002) validated in the Spanish context by Moreno-Murcia and Conte (2011) was used. The scale consists of 25 items, grouped into five dimensions: Fear of experiencing embarrassment (7 items; e.g., *“When I make a mistake, I am embarrassed if others are there to see it”*), Fear of self-devaluation (4 items; e.g., “*When I am unsuccessful, I feel less valuable than when I am successful”*), Fear of having an uncertain future (4 items; e.g. *“When I am wrong, I think my plans for the future will change”*), Fear of losing important others’ interest (5 items; e.g.,” *When I am not successful, some people are not interested in me”*), and Fear of upsetting important others (5 items; e.g., *“When I am wrong, it upsets people I care about”*). Each item began with the statement, “*In practicing my sport....”* Also, this measure has shown a good fit to use a General Factor denominated Fear of Failure that will be used in our results. Responses were collected on a 5-point Likert-type scale ranging from I do not believe it at all (1) to I believe it 100% (5). Previous studies evidenced good psychometric properties, including support for factorial, external, and predictive stability validity (e.g., Moreno-Murcia and Conte, 2011). In this study, the internal consistency analysis has been satisfactory, showing a CFA (χ2/gl = 34.36; TLI = 0.93; CFI = 0.95; SRMR = 0.04; RMSEA = 0.06) and a value of Cronbach’s alpha = 0.95.

Perfectionism. A Spanish version short form of the Multidimensional Inventory of Perfectionism in Sport (MIPS; Stoeber et al., 2007), adapted to the Spanish context by Atienza et al. (2020) was administrated. It comprises five items that capture Negative Reactions to Imperfection (NRI) (e.g., “I feel extremely stressed if everything does not go perfectly”) and five items that measure individual differences in the Pursuit of Perfection (Perfectionistic Efforts; PE) (e.g., *“I strive to be as perfect as possible”*). The items were preceded by the root *“When I play or practice basketball.”* Participants responded to the items on a scale ranging from 1-"never” to 6-"always.” The reliability found for the sample showed a fit CFA (χ2/gl = 45.1; TLI = 0.92; CFI = 0.96; SRMR = 0.03; RMSEA = 0.08) and a Cronbach’s alpha of 0.87.

### 2.3 Procedure

Data collection was carried out using a questionnaire developed *ad hoc* for this study on the Google Form® platform, with two validated scales composed of questions formulated in a clear and precise manner to obtain truthful information from the respondents. The questionnaire was administered online in January, February and March 2024. All participants were informed of the objective of the study and of their rights as participants in the study, as well as of the voluntary nature of the study, the absolute confidentiality of the answers and the treatment of the data, and that there were no right or wrong answers, and they were asked to answer with the utmost sincerity and honesty. The average time taken to complete the questionnaire per participant was 15 min. After verification of the data, the following variables included in the questionnaire were recorded: age, sex, refereeing category, refereeing experience, previous refereeing experience in handball, whether or not they are referees in another sport and whether or not they have been promoted The link to the questionnaire was as follows: https://tinyurl.com/4fpbp7ha. This study was carried out in accordance with the Declaration of Helsinki (World Medical Association, 2013) and is part of the research project entitled “Analysis of the factors implicit in the teaching-learning process of the handball player,” which obtained a favorable report from the Research Ethics Committee of the University of Murcia (ID: 4447/2023).

### 2.4 Statistical analysis

First, the sample was purified by outlier analysis with the Mahalanobis distance where a total of 2 subjects were eliminated (value < 0.01) after incorporating the variables under study and descriptive statistics were performed taking into account the skewness and kurtosis indices, looking for values < 1.96 according to Field (2017), which indicates similarity with the normal curve in a univariate way. Next, the reliability of each of the scales was calculated using Cronbach’s alpha and, finally, the correlations between the subscales were calculated using Spearman’s correlation, homogeneity of variances, and after checking the normality of the data, adjustment tests correspondently (Kolgomorov-Smirnov, internal reliability, and Cohen’s), and differential analyses (*U*-ManWittney) for the mean difference. Also, multivariate analysis of variance (Kruskal Wallis) was performed in which the variables sex, years refereeing, and type of experience were controlled. Finally, multiple linear regression and interaction model (*p* < 0.05; bootstrap sample = 5,000). All statistics were performed with the IBM SSPS 25.0 program.

## 3 Results

### 3.1 Descriptive

The trends on perfectionism and fear of failure are shown to differ under sex differences in favor of females (see Table 2) in the negative reactions of imperfection. In the previous referee experience, the Kruskal-Wallis test showed 3 groups [Never in other sporting roles, player and coach, and only player] (*X^2^* = 3.482; <0.04).

**Table 2:** Differential test (independent samples), non-parametric data.

	Group	Levene	*N*	Mean	SD	M-Wittney	*p*	Effect size
Striving for perfection	Male	0.11*	95	4.352	1.044	1005.500	0.09	−0.216
	Female		27	4.711	1.032			
Negative reactions to imperfection	Male	0.55*	95	3.320	1.096	836.500	0.01*	−0.348
	Female		27	4.037	1.249			
Fear of failure	Male	4.97	95	2.124	0.888	1022.000	0.11	−0.203
	Female		27	2.519	1.104			

*p < 0.05.

### 3.2 Correlations and regression analysis

**Figure 1:**
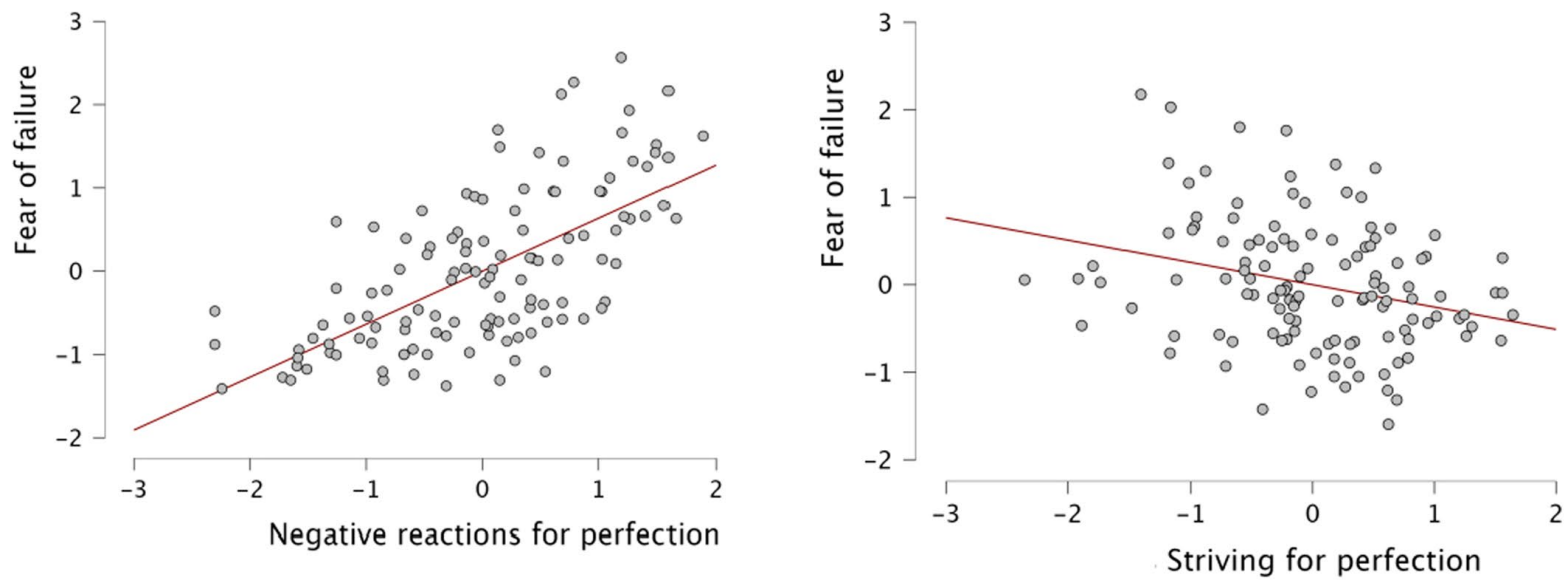
Regression analysis.

The relationships between variables are significantly positive when controlling for both sex and previous referee experience (see Table 3). It is important to highlight in both analyses the negative relationships between the desire for perfection and the fear of failure, while the meanings are positive between the fear of failure and the negative reactions to imperfection. The regression models (stepwise) offer different adjustments to explain the variance (see Table 4 and Figure 1) of the prediction of fear of failure from the perfectionist tendencies in the sample of referees analyzed.

**Table 3:** Spearman correlation analysis, controlling gender and previous experience.

	Range	1	2	3
1. Striving for perfection	1–6	–		
2. Negative reactions to imperfection	1–6	0.587^**^	–	
3. Fear of failure	1–5	−0.189*	0.622^**^	–

^*^si *p* < 0.05; ^**^si *p* > 0.01.

**Table 4:** Regression analysis models.

**Model**	***R***	***R*** ^**2**^	**Adjusted *R*** ^**2**^	**RMSE**	***R***^**2**^ **Change**	***F* change**	**df1**	**df2**	***p***
1	0.000	0.000	0.000	0.949	0.000		0	121	
2	0.622	0.387	0.382	0.746	0.390	75.851	1	120	<0.001*
3	0.659	0.435	0.425	0.720	0.481	10.015	1	119	<0.002*

*p < 0.05.

**Figure 2:**
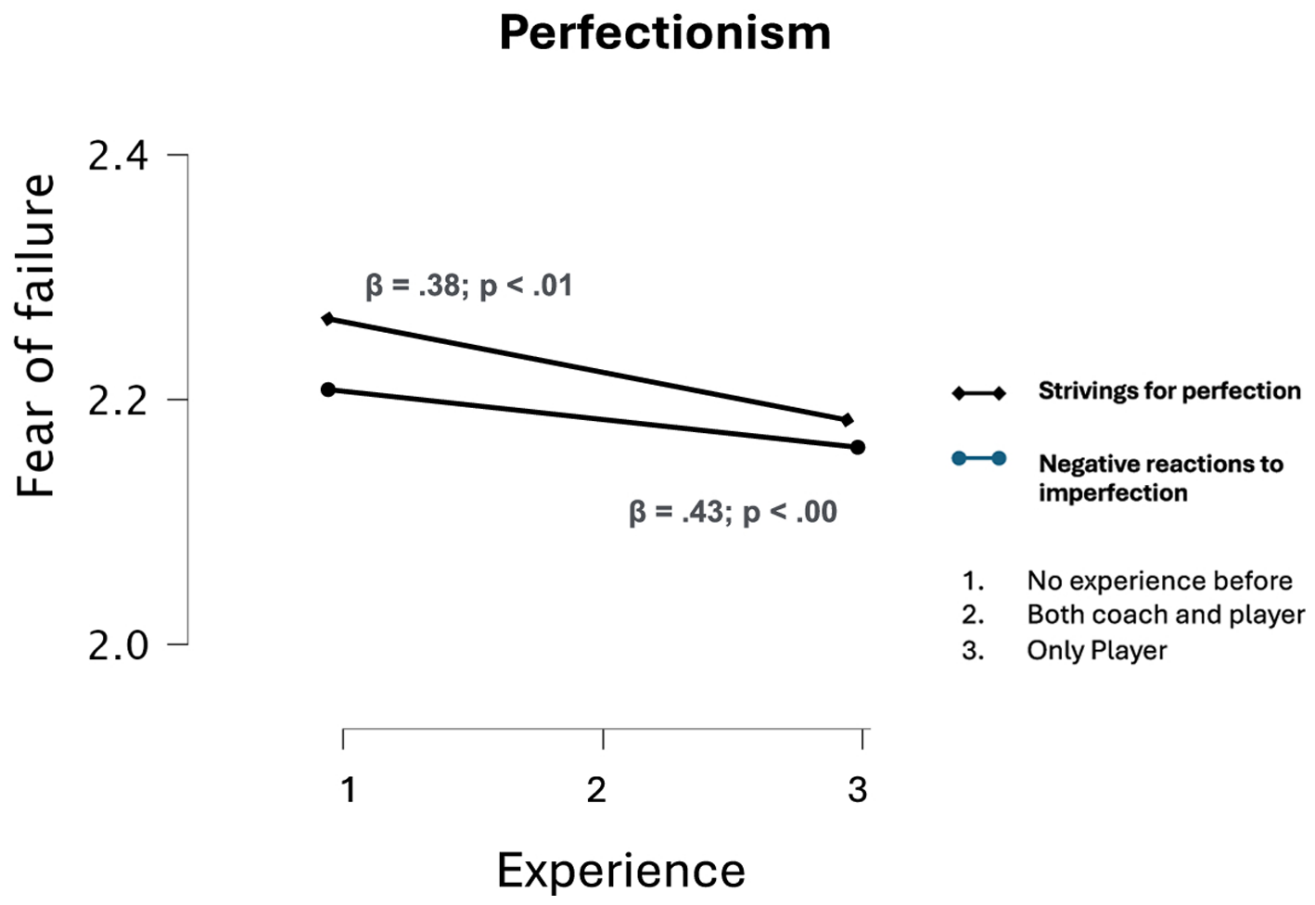
Analysis of interaction effects by experience.

As it can be seen, both perfectionist tendencies and fear of failure offer statistically significant interaction effects when sports experience before becoming a referee are compared (see Figure 2), controlling the impact of sex differences (*p* < 0.05).

## 4 Discussion

The aim of the study was to describe the effects that the previous sports experiences of handball referees had on the relationships with perfectionism and fear of making mistakes. The results of the differential analysis showed the existence of statistically significant differences in negative reactions to imperfection according to sex (in favor of women) and according to previous sports experience (in favor of referees who were coaches or handball players before becoming referees). However, the previous experience of referees as well as other figures in the sports situation (mainly having been players) has shown a moderating effect for the reduction of the negative relationship between perfectionism and fear of failure (mainly from negative reactions to imperfection).

As mentioned in the introduction, the results of this work (also in referees) insist on the controversy about the maladaptive nature of perfectionism proposed in classical literature (e.g., Egan et al., 2011; Flett and Hewitt, 2002; Stumpf and Parker, 2000). Either these are vulnerability processes that impair the athlete’s performance (Flett and Hewitt, 2005), or an adaptive trait (e.g., Rasquinha et al., 2014; Rice et al., 2012; Stoeber et al., 2014), that favor the achievement of good sport performance (Gould et al., 2002). Hill et al. (2015) pointed out that it is a source of inner drive that provides greater capacity for fulfilment, but at the same time also subjects people to more tension in their lives, and that in the long-term, it has adverse effects on both the mental health and performance of people who are performance-oriented.

Concerning to the sex of the participants, there is an interesting debate from studies carried out in other contexts. We can find studies referred to samples of different ages (adolescents and university students) (e.g., Efek and Türegün, 2022; Hassan et al., 2012; Hewitt and Flett, 1991; Vuyk, 2015) and others in which was found that men reflected the highest levels of perfectionism (Jara-Moreno et al., 2020) where non-statistically significant results were found. A similar result was obtained in the present study with referees. In general, the literature focused on sports indicates that women reflect the highest values in negative reactions to imperfection (similar to the results found in the general population) and men in efforts to be perfect, mainly if they focus on competitiveness and high performance.

In terms of experience, the literature has shown that those referees with more years in the role of referee achieve better psychological characteristics (Muñoz-Arjona et al., 2022). The results of the study cannot be compared since the rest of the research has treated this variable as years of refereeing (e.g., Muñoz-Arjona et al., 2022) or competitive level (e.g., Guillén and Feltz, 2011), without considering the previous experience of the referees before performing this role and their positive transfer in their training.

To achieve the proposed objective, correlation and regression analysis were carried out controlling both analyses for sex and previous experiences of the referees. Correlation analysis reflected a negative relationship between perfectionist efforts (e.g., high personal standards, attention to detail) (Frost et al., 1990; Stoeber and Otto, 2006; Stumpf and Parker, 2000) and the fear of making mistakes. On the other hand, a positive relationship is shown between negative reactions to the imperfection of referees (e.g., perfectionist concerns, self-devaluations of their effectiveness) associated with excessive fear of making mistakes (Frost and Henderson, 1991; Hill et al., 2004). The results are consistent with those found in previous studies that have analyzed the relationship between both variables (e.g., Koivula et al., 2002; Stoeber et al., 2007) and support the distinction between the two dimensions of perfectionism in sport, as theorized by different authors (e.g., Frost et al., 1990; Hewitt and Flett, 1991; Stoeber and Otto, 2006).

Literature has shown that people who have high levels of striving for perfection can show high levels of negative reactions to imperfection, maintaining such high expectations about how they expect things to turn out, that they may experience a persistent fear of not living up to those standards that they have self-imposed. Often so high that they are unrealistic and connected to the negative consequences of not achieving them (e.g., failing teammates or coaches, feeling embarrassed) (e.g., somatic and cognitive anxiety before competition) (Pineda Espejel et al., 2017). If it is already common in human behavior not to make mistakes and/or the fear of not achieving their goals, those people who show greater tendencies towards perfection exaggerate the constant avoidance of challenging situations, even if they are motivating and positive. Finally, regression analysis showed that both perfectionist tendencies and fear of failure offer statistically significant interaction effects on fear of failure.

Having obtained results that contrast, add and corroborate others previously obtained by other studies, we need to recognize some limitations which show the present study. As the scarce existing literature focused on the analysis of perfectionism and emotional or cognitive responses in the figure of the referee. Other limitations which should be considered for future studies, could be focused in analyzing whether the referee act alone or in pairs. In addition, the denomination of federated categories depends on Territorial Federations. That question would difficult the design of longitudinal studies which could detect the regular changes in the referee’s careers (e.g., assistants, VAR, important matches).

On the other hand, findings should be seen as preliminary and will need to be replicated in future studies with a larger and more varied sample, thus covering all federation categories and different referee profiles. The present study advances the theoretical implications of the explanation of the fear of making mistakes in sport, since it has been an effort to better understand the modulators in the relationship between perfectionist tendencies and the fear of failure in sport. In addition, the fact that the study focuses on referees, even if they are only from a specific sport, inspires to deepen them not only by increasing the number of studies but also by offering greater quality to the interpretations when they infer aspects linked to personality such as the tendency to perfectionism and the externalization of attention to detail in sports action.

Precisely, the figure of referees involves the administration of the justice of the rules for each sport, with the responsibility that this entails and with the repercussions that they entail for any figure involved in the game (e.g., players, coaches, supporters). On a practical level, the results have implications for referees, because they show that, even with efforts to improve behaviors and decisions, the training of psychological skills (e.g., positive cognitive regulation towards understanding error, stopping negative self-verbalizations) and understanding of personal circumstances (e.g., previously acquired experience) will allow the impact of their tendencies towards the pursuit of perfection (sometimes already necessarily understood to improve actions, compete with other arbitrators or apply adequate processes of self-efficacy). All these can be sources of exhaustion, constant frustrations, and coexistence with criticism (internal and external), resulting in a wear and tear that will do little to help them adapt to sports situations.

It is worth highlighting in terms of the strengths of this study, on the one hand, the innovation in the subject investigated, since, as has already been mentioned on several occasions, referees are an object of study on which there has been little research done. That is why it has been decided to study them, in order to open the way to future research in this field and, specifically, in handball referees. Another strength would be to analyze the fear of making mistakes and perfectionist tendencies since we have found few previous studies that have analyzed them under this approach, as well as that has considered the different variables related to arbitration that have been included in this study.

## 5 Conclusion

The results of the study showed differences in perfectionist tendencies according to gender, in favor of women and based on previous sports experience, in favor of referees who were coaches or handball players before becoming referees. It was also shown that both perfectionist tendencies and fear of failure offer interaction effects on fear of failure.

In the focus to work in psychological trainings with referees samples, the evaluation of this type of variables allows the design of prevention and health intervention programs and/or stimulation of adaptive coping strategies, emotional self-regulation skills, which contribute to referees with high levels of fear of failure or socially prescribed perfectionism, since there is a strong positive relationship between both variables, are competent to deal effectively with stressful situations prior to competition.

Finally, it would be interesting to make room for future research in this area that is studied so little, since it could help to maximize the performance and improve the mental health of referees, in this case handball. This would lead to a substantial improvement in the quality of the different competitions that take place in our country, from the training category to professional competitions. To do this, the different profiles of referees that may arise depending on the sport modality (individual or team sports) could be analyzed and compared.

## Data Availability

The raw data supporting the conclusions of this article will be made available by the authors, without undue reservation.
